# Laser Direct Writing via Two-Photon Polymerization of 3D Hierarchical Structures with Cells-Antiadhesive Properties

**DOI:** 10.3390/ijms22115653

**Published:** 2021-05-26

**Authors:** Irina A. Paun, Bogdan S. Calin, Cosmin C. Mustaciosu, Eugenia Tanasa, Antoniu Moldovan, Agata Niemczyk, Maria Dinescu

**Affiliations:** 1 Center for Advanced Laser Technologies (CETAL), National Institute for Laser, Plasma and Radiation Physics, RO-077125 Magurele-Ilfov, Romania; bogdan.calin@inflpr.ro; 2 Faculty of Applied Sciences, University Politehnica of Bucharest, RO-060042 Bucharest, Romania; eugenia.tanasa@physics.pub.ro; 3 Horia Hulubei National Institute for Physics and Nuclear Engineering IFIN-HH, RO-077125 Magurele-Ilfov, Romania; cosmin@nipne.ro; 4 Faculty of Applied Chemistry and Materials Science, University Politehnica of Bucharest, RO-060042 Bucharest, Romania; 5 National Institute for Laser, Plasma and Radiation Physics, RO-077125 Magurele-Ilfov, Romania; antoniu.moldovan@inflpr.ro (A.M.); maria.dinescu@inflpr.ro (M.D.); 6 Department of Materials Technology, Faculty of Mechanical Engineering and Mechatronics, West Pomeranian University of Technology in Szczecin, 19 Piastow Ave, 70-310 Szczecin, Poland; aniemczyk@zut.edu.pl

**Keywords:** hierarchical structures, laser direct writing via two-photon polymerization, wettability, cell adhesion

## Abstract

We report the design and fabrication by laser direct writing via two photons polymerization of innovative hierarchical structures with cell-repellency capability. The structures were designed in the shape of “mushrooms”, consisting of an underside (mushroom’s leg) acting as a support structure and a top side (mushroom’s hat) decorated with micro- and nanostructures. A ripple-like pattern was created on top of the mushrooms, over length scales ranging from several µm (microstructured mushroom-like pillars, MMP) to tens of nm (nanostructured mushroom-like pillars, NMP). The MMP and NMP structures were hydrophobic, with contact angles of (127 ± 2)° and (128 ± 4)°, respectively, whereas flat polymer surfaces were hydrophilic, with a contact angle of (43 ± 1)°. The cell attachment on NMP structures was reduced by 55% as compared to the controls, whereas for the MMP, a reduction of only 21% was observed. Moreover, the MMP structures preserved the native spindle-like with phyllopodia cellular shape, whereas the cells from NMP structures showed a round shape and absence of phyllopodia. Overall, the NMP structures were more effective in impeding the cellular attachment and affected the cell shape to a greater extent than the MMP structures. The influence of the wettability on cell adhesion and shape was less important, the cellular behavior being mainly governed by structures’ topography.

## 1. Introduction

One of the major challenges in bioengineering is to develop technological approaches that provide maximum control over cellular adhesion [1]. Cell-repellent surfaces are particularly useful in cell-based biosensing for drug delivery systems, as well as for biomedical implants and tissue engineering [1]. A key strategy for controlling the cellular attachment is related to the fact that all cell types are influenced by spatial elements (geometry and size) of cell culture substrates. More precisely, cells are influenced by the width, spacing, and feature depth of these substrates [2]. Until now, a broad range of micro and nanopatterns in the form of grooves, pillars, cones, channels, and other micro/nano-geometries have been developed for controlling the cellular attachment [3,4,5]. A plethora of studies report on the combined roles of surface topography and wettability of a surface on cellular adhesion [6,7]. The joint influence exerted by different surface topographies and wettability, obtained using various processing methods and formulations, on the cell adhesion have been tested [8,9].

Examples from nature indicate that the most efficient substrates for repelling living organisms are those with multiple size scales that act in parallel [10]. In particular, hierarchical structures possessing elements of different length scales that overlap with one another have proved efficient for preventing the settlement of organisms of different sizes [10,11]. Hierarchical topographies comprising nano- and microscale structures have been shown to influence the behavior of various cell types, including endothelial cells, osteoblasts, neural phenotype cells, and stem cells [8,11].

Hierarchical structures have been fabricated by top-down, bottom-up, and hybrid approaches [12,13,14]. The top-down methods such as nanoimprint lithography, soft lithography, and capillary force lithography require additional efforts such as the application of pressure, heat, or coating the surface of the substrate with a thin adhesive layer that overwhelms the adhesion between the imprint mold and the patterned layer. The bottom-up methods (e.g., self-assembly) provided more complex 3D biomimetic structures, but they were less accurate because of uncontrollable parameters such as the chemical and physical states of the surface (including defects). For hybrid methods merging top-down and bottom-up approaches, such as pre-patterning and post-structuring or pre-masking and post-structuring, it is difficult to guide the deposition/growth of nanomaterials on pre-defined locations on micrometric structures. To sum up, the existing technologies involve rather complicated, multi-step procedures; the development of multiscale, hierarchical structures without the use of sophisticated equipment and complex processes remains challenging [15].

In this study, we demonstrate a simple, single-step method based on laser direct writing via two photons polymerization (LDW via TPP) of a photopolymerizable material for the fabrication of innovative hierarchical structures to act against the adhesion of glial cells. LDW via TPP is a 3D printing technology based on additive manufacturing that uses a computer-controlled design to obtain complex 3D architectures in photopolymerizable materials [16,17]. It can be used to obtain structures with spatial resolution as low as 90 nm and provides fully reproducible geometries, with practically no constraints concerning the achievable architectures [17,18]. Presently, LDW via TPP is used for the fabrication of a broad variety of 3D micro- and nanostructures for tissue engineering and regenerative medicine applications [19,20,21]. We recently demonstrated the versatility of LDW via TPP for fabricating complex 3D microstructures able to guide the attachment of osteoblast and fibroblast cells [22,23,24]. The innovative hierarchical structures developed in the present work have two levels of structuring: the first level consisted of micrometric mushroom-like constructs, and, in the second level, the mushrooms’ hats were “decorated” with micro- and nanostructures in the shape of elliptical ripple-like patterns. The obtained hierarchical structures were further denominated microstructured mushroom-like pillars (MMP) and nanostructured mushroom-like pillars (NMP), respectively. The MMP and NMP structures were separately arranged in hexagonal lattices laying on hundreds of squared micrometers. The roles exerted by the topography and the wettability of the obtained surfaces were investigated in vitro regarding the shape and the adhesion behavior of glial cells. We particularly addressed the roles of the nano- and microstructuring and of the wettability of the MMP and NMP hierarchical structures in preventing cellular adhesion.

## 2. Results and Discussion

### 2.1. Design of Hierarchical Structures

The design of repellent surfaces aimed to minimize the break-in force exerted by the pressure of the liquid that wets the structure and to maximize the surface tension that suspends the liquid and prevents wetting; these constructs relied on doubly re-entrant structures consisting of microscale posts with nanoscale vertical overhangs [25]. As an alternative to the traditional “overhangs”, in this study, we propose a novel hierarchical structure in the shape of a mushroom-like pillar where the mushroom’s “hat” was decorated with micro- and nanostructures, respectively.

**Figure 1:**
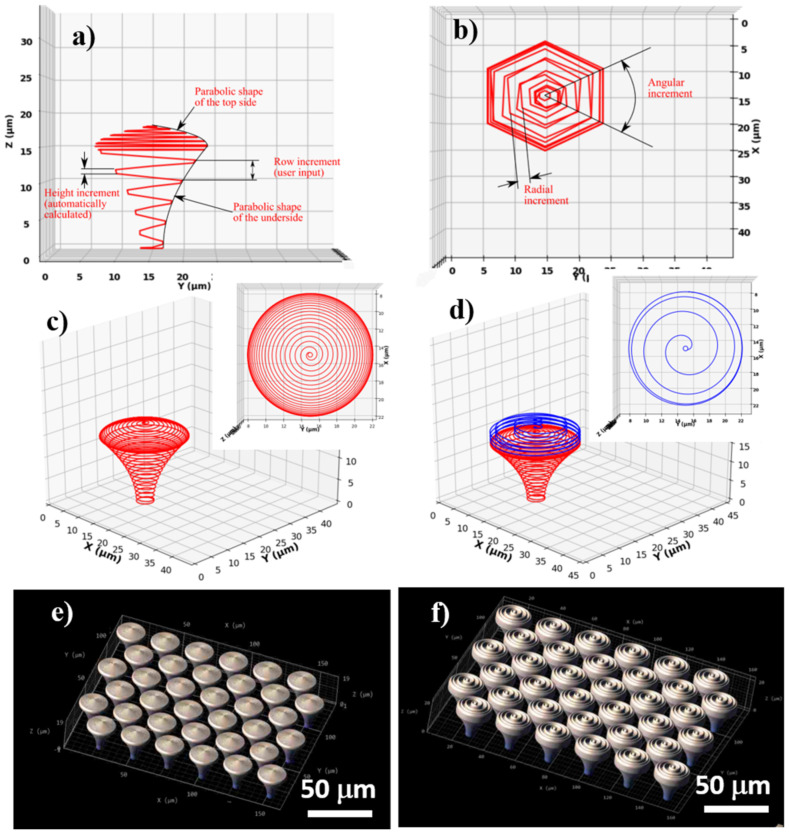
Schematic representation of a mushroom-like pillar designed using an iterative algorithm based on the equation of an ellipse: (**a**) lateral view; (**b**) top view. Design of mushroom-like pillars: (**c**) nanostructured mushroom-like pillar (NMP); (**d**) microstructured mushroom-like pillar (MMP) (tilted views). The insets illustrate top views of mushrooms’ hats; (**e**,**f**) simulation of the mushroom-like pillars from (**c**) and (**d**), arranged in hexagonal lattices, using DeScribe dedicated software of Nanoscribe 3D-lithography system.

For structure design, the 3D lithography Nanoscribe installation requires an ordered list of Cartesian points that describe the path followed by the voxel during the laser writing process. We designed hierarchical structures based on an iterative algorithm that calculated the coordinates of each point of the structure consecutively and where the laser path followed a spiral shape. To evaluate the Cartesian coordinates of each point, the equation of an ellipse was used. For the following point, the parameters of the equation were each modified with a specific incremental value (radius, height, and angle). In order to obtain structures with circular symmetry in the XY plane, an angular increment of 3° between consecutive points was employed. The software was written such that the user inputs the total height of the structure and the height step of consecutive spirals (row increment). As such, radial and height increments were automatically calculated to accommodate the given geometric characteristics. The concept of the design is illustrated in Figure 1a,b.

The structure is defined by two parts, from the design point of view: the underside, or the mushroom’s leg acting as a support structure, with a surface curved toward the vertical symmetry axis of the pillar, and the top side (the mushroom’s hat), with a surface curved away from the vertical symmetry axis of the pillar. The underside and top side of the mushroom-like structures have a parabolic-shaped surface, in the vertical transverse section that goes through the center. The parabolic curvature of the mushrooms’ legs and hats has the role of minimizing the cell-substrate contact area.

For calculating the appropriate Cartesian coordinates, a parabolic equation for each part was initially defined, then it was scaled down to fit the user inputted sizes. The radius is determined by:

(1) $$r=r_{top}+\frac{\left(z^{2}+exp\left(11-\epsilon \right)\cdot z\right)\cdot \left(r_{top}-r_{bottom}\right)}{\left(h^{2}+exp\left(11-\epsilon \right)\cdot h\right)}$$

for the underside, and:

(2) $$r=r_{top}+\frac{0.2\cdot \left(z^{2}+exp\left(\epsilon \right)\cdot z\right)\cdot \left(r_{top}-r_{bottom}\right)}{\left(h^{2}+exp\left(\epsilon \right)\cdot h\right)}$$

for the top side, where:

*r* = structure radius as a function of height
*r_bottom_* = minimum radius
*r_top_* = maximum radius
*ϵ* = a curvature coefficient (constant value); for *ϵ* = 0 a conical shape is obtained
*z* = height
*h* = maximum height (i.e., 15 µm for the underside and 3 µm for the top side)

Using the above-described algorithm, we designed and fabricated arrays of mushroom-like pillars placed in a hexagonal lattice. The pillars had a total height of 18 µm, of which 15 µm was the supporting underside structure, and 3 µm was the top side, each of these parts having different parabolic curvatures. The diameter of the base was 4 µm, the diameter of the top was 20 µm, and the row height was 1 µm for the underside and 0.2 µm for the top side. The difference in the row height for each part of the pillar (i.e., underside and top side) was determined by the shape of the voxel, which is a prolate volume of approximately 2 µm height and 1 µm width. This was performed in order to achieve an appropriate overlap of neighboring voxels that provide suitable mechanical stability for the structures. It is worth mentioning that the height increment was constant, but the radial increment varied to accommodate the curved surface.

For a set of structures (denominated microstructured mushroom-like pillars—MMP), a spiral wall on the outer surface of the top side was added (i.e., on the mushroom’s hat) (Figure 1d). The spiral followed the parabolic surface of the top side, but the radial step was significantly increased in order to obtain separated walls. In order to separate the walls of the spiral, the row height was increased to 6 times the row height of the top side (i.e., to 1.2 µm). For both MMP and NMP structures, the topographical gradients provided by variable spiral steps over the hats of the mushroom-like structures are expected to provide an efficient means to control the degree of overlapping in order to obtain a high control over the aspect ratio of the micro- and nanostructures.

### 2.2. Fabrication of Hierarchical Structures

**Figure 2:**
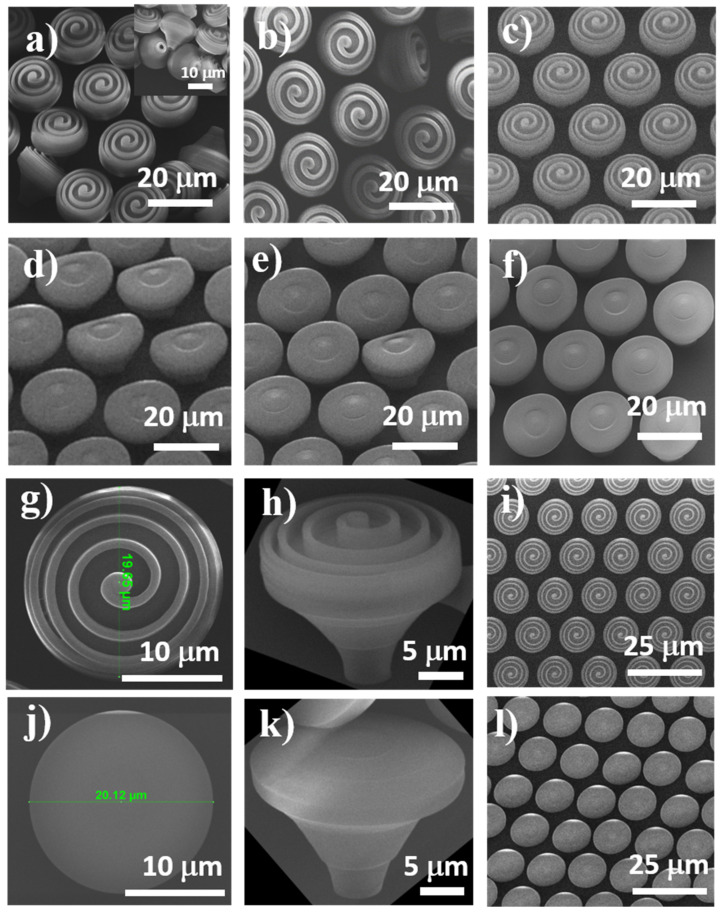
Scanning electron micrographs of microstructured (MMP) and nanostructured mushroom-like pillars (NMP) fabricated by LDW via TPP with various laser writing parameters: (**a**,**d**) laser speed 140 µm/s, laser power 13.8 mW; (**b**,**e**) laser speed 120 µm/s, laser power 12.5 mW; (**c**,**f**) laser speed 100 µm/s, laser power 12.5 mW. MMP and NMP structures fabricated using laser speed 100 µm/s and laser power 13.8 mW: (**g**–**i**) MMP (**j**–**l**) NMP; (**g**,**j**) close, top views of mushrooms’ “hats”; (**h**,**k**) close, tilted view of single MMP and NMP, respectively; (**i**,**l**) large, top views of MMP and MNP areas, respectively.

Morphological investigations of the laser-fabricated structures (Figure 2) indicate that they fully reproduced the design from Figure 1. First, we performed a parametric study in order to find the best experimental conditions for fabricating the structures. Laser scanning speeds between 80 and 120 µm/s and laser powers between 10 and 14 mW were used. The lower limit of the laser power was the one that allowed material to photopolymerize (as observed by visual inspection using the camera of the 3D lithography system); the upper limit was the one that induced local micro-explosions of the photopolymerizable material, likely due to local overheating. Based on similar considerations, the lowest value of the laser scanning speed corresponded to the threshold photopolymerization, while the upper limit was the one above which micro-explosions occurred.

Rather than discussing the laser scanning speed and laser power as independent parameters, the morphology of the laser-fabricated structures was further analyzed in terms of irradiation dose arising from a combination between the laser scanning speed and the laser power. For relatively high laser scanning speed and laser power, i.e., 140 µm/s and 13.8 mW, the micro- and nanostructures form the top of MMP and NMP structures were rather irregular. Both MMP and NMP structures showed poor stability on the glass substrate (Figure 2a,d). Some of the MMP collapsed during and after development, but they preserved at a great extent their original shape (Figure 2a), while some of the NMP were inclined and were undulated on the top (Figure 2d). This mechanical instability could be tentatively ascribed to different factors. One is an insufficient polymerization degree of the photopolymerizable material, leaving behind traces of unpolymerized monomers in and around the structures that were washed away during the developing process and destabilized the structures. Another reason could be that the top of the MMP structures were much heavier than the base because of the extra-spiral from the top of the mushrooms that induced a mechanical disequilibrium in the structures. The fact that the NMP (structures not having the large spiral on top) maintained their vertical position and only a few of them were slightly bent (Figure 2d) sustains this hypothesis. The third possible reason for structures’ mechanical instability is that the irradiation dose resulted from the combination of laser speed and laser power was probably not sufficient to polymerize the whole volume of the photopolymer from the inner parts of the mushroom-like structures. Thus, the mushrooms’ hats and posts remained filled with a liquid photoresist that was washed away during the developing process; the liquid photoresist from the contact points between the mushroom-like structures and the glass surface most likely favored the mechanical instability of the structures that collapsed (as observed for MMP) or inclined (as observed for NMP). An additional argument for this hypothesis is provided by the inset from Figure 2a, indicating that after the developing process, the MMP were flipped over, but they preserved their geometrical shape; it is interesting to note that the external walls of the mushroom-like structures were polymerized, whereas the inner parts were empty cavities that most probably remained after the unpolymerized material from the inner part of the structures was washed away.

When decreasing the laser speed to 120 µm/s and the laser power to 12.5 mW, some of the MMP structures still had poor stability on the substrate and collapsed (Figure 2b). For NMP structures, the top of the mushroom-like structures showed weak undulations, and the ripples were inhomogeneously distributed (Figure 2e). This occurred most probably because the laser irradiation dose was still too low to achieve a complete photopolymerization.

Upon decreasing the laser speed to 100 µm/s while maintaining the laser power at 12.5 mW, both MMP and NMP structures showed suitable stability on the substrate. They fully reproduced the design, and the elliptical ripple-like features decorating the mushrooms’ hats were homogeneously distributed (Figure 2c,f). These experimental findings indicate that this specific interplay between the laser power, laser speed, and design, which in turn govern the voxels size and overlapping, enable the photopolymerization of the whole volume of irradiated photopolymer and provided suitable mechanical stability for the structures. Based on the above experimental considerations, the optimum laser parameters used for further fabrication of the hierarchical structures were identified to be 100 µm/s for the laser scanning speed and 12.5 mW for the laser power.

**Figure 3:**
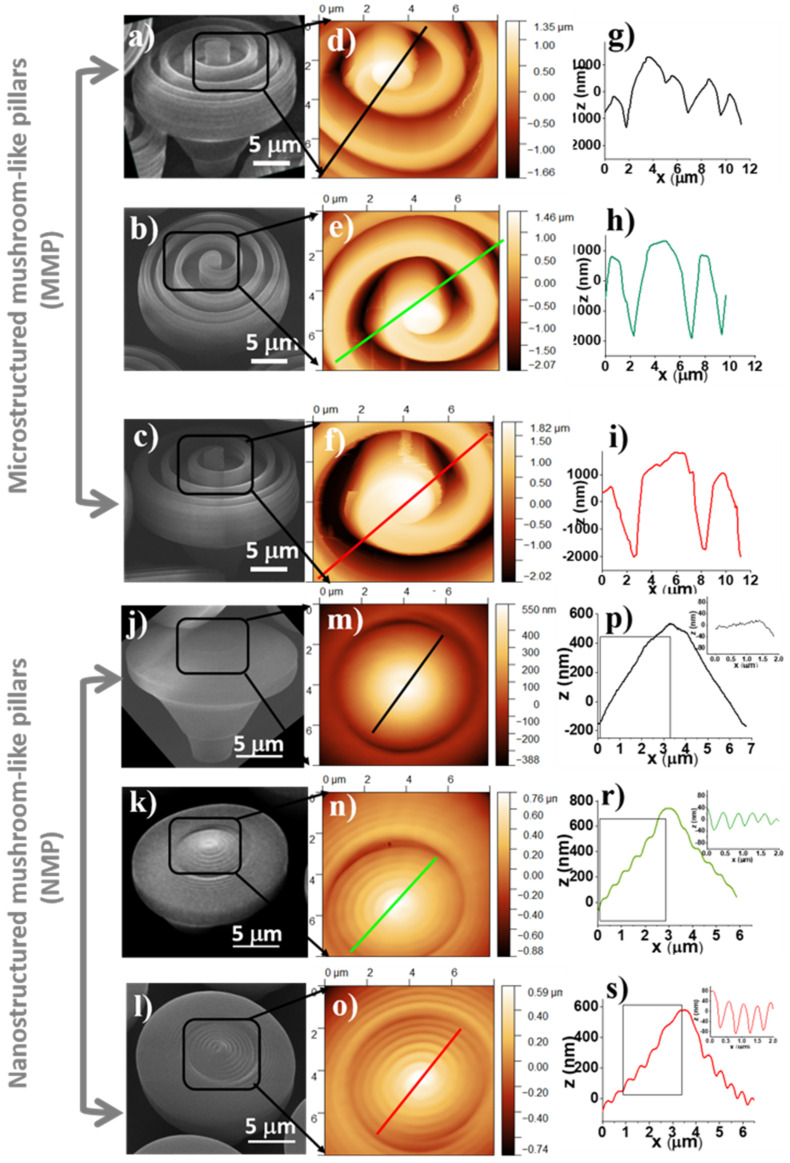
Scanning electron micrographs (**a**–**c**,**j**–**l**), atomic force microscopy images (**d**–**f**,**m**–**o**) and line profiles measured from AFM images (**g**–**i**,**p**–**s**) of micro- and nanostructured mushroom-like pillars (MMP and NMP). Laser writing parameters: (**a**,**j**) laser speed 140 µm/s, laser power 13.8 mW; (**b**,**k**) laser speed 120 µm/s, laser power 12.5 mW; (**c**,**l**) laser speed 100 µm/s, laser power 12.5 mW.

AFM measurements were also performed to obtain a quantitative insight into the 3D topography of the MMP and NMP. Joint SEM and AFM images of micro- and nanostructured mushroom-like pillars (MMP and NMP) fabricated by LDW via TPP using various combinations of laser scanning speeds and laser powers are presented in Figure 3: SEM (Figure 3a–c,j–l) and AFM (Figure 3d–f,m–o). Particular attention was dedicated to the periodic micro- and nanostructures from the top of the mushroom-like pillars that were measured in respect to depth and width through 2D profiles from 3D AFM images (Figure 3g–i,p–s).

In Table 1 are presented heights, periodicities, and aspect ratios of the ripple-like features decorating the top of the NMP and MMP structures measured based on the line profiles depicted in Figure 3. For MMP structures, the heights of the periodical ripple-like features ranged between 2540 and 3800 nm, and the width ranged from 1734 up to 5667 nm, both depending on the design and the laser power and scanning speed. The aspect ratio, defined as the ratio between the width and the height of the periodical features, ranged between 1.3 and 1.5. To note that for the MMP structures fabricated using laser power of 13.8 mW and laser scanning speed of 140 µm/s, the AFM needle could not reach the bottom of the structures, i.e., the spiral walls were too high, and the height of the spiral-like microstructures could not be measured. For the NMP structures, the ripple-like features from the top of the mushrooms were between 69 and 155 nm in height, and their periodicities ranged between 440 and 740 nm, with calculated aspect ratios between 3.8 and 7.2.

The side walls of the MMP top structures are basically vertical, as shown in the SEM images (Figure 3a–c). Considering also the large sizes and aspect ratios of these structures, their imaging by AFM was performed at a relatively low scanning rate (0.1 Hz) in order to allow a suitable tracing of the steep topography profiles while still maintaining the tip-sample interaction as low as possible. For the same reason, relatively large gain values were necessary. The tip oscillation amplitude was held at 7–8 nm (nominal, uncalibrated values). In this respect, the NMP structures presented no particular difficulty in AFM imaging.

**Figure 4:**
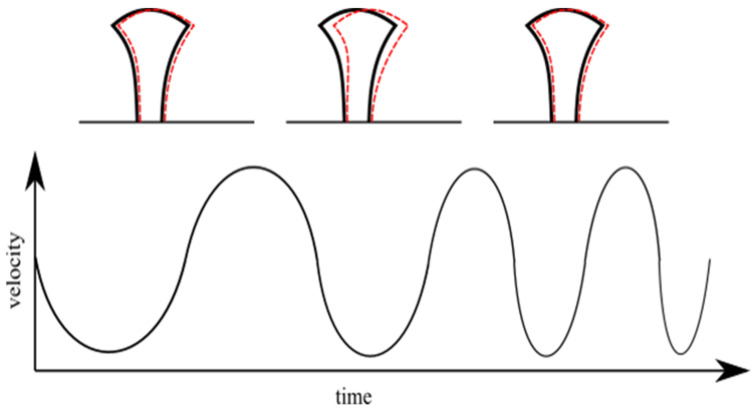
Schematic representation of the resonant oscillations of the mushroom-like pillars determined by the stage movement.

It can be noticed (see SEM and AFM images in Figure 3) that the nanostructured mushroom-like pillars (NMP) show some structural defects at the top, in the shape of an indentation in the middle of each structure. These defects have a high reproducibility (they were observed on each pillar), with similar geometric characteristics, i.e., same diameter and asymmetry. These could be determined by polymer shrinkage following sample development procedure; however, this hypothesis would not explain the high reproducibility of the defects. Another hypothesis is related to the fabrication method, i.e., stationary laser beam and moving sample. More specifically, the mushroom-like pillars were written using a continuous spiral, which means the structures were moved in a quasi-circular pattern throughout the writing process (quasi-circular due to the small radius increment). This moving pattern could have induced oscillations of the pillars, especially since the mushroom-like pillars were top-heavy with slim support. The writing speed was constant throughout the writing of a pillar, which means each axis of the translation stage moved back and forth, with a continuously variable frequency and a sinusoidal acceleration. The variable movement frequency was determined by the variable radius of the spiral. This suggests that the stages movement and the pillar oscillation might have entered in a resonance process for a short time (due to the continuously variable movement frequency of the stages). This could explain both the reproducibility of the indentation as well as its asymmetry. The hypothesis is schematically represented in Figure 4.

In recent years, the control of cellular shape and attachment using polymeric nano- and microstructures such as grooves, pillars, ridges, pores, and wells attracted great interest in tissue engineering and applied biomaterials research [26]. Micro- and nano-fabrication techniques, including photolithography, electrospinning, and laser structuring, have been developed for precise surface patterning of various materials such as polymers, ceramics, or metals, at the cellular and the subcellular scale [8,27]. Particular attention is currently dedicated to hierarchical structures consisting of different levels of structuring at micro- and nano-scales, which showed potential for modulating the attachment of living organisms, including cells. To date, hierarchical structures have been developed by micropattern fabrication followed by the growth of nano-topographical features, but these approaches required multi-step, complicated procedures and provided poor control over the resulting architectures [9,13,28].

In the last decade, laser-assisted patterning has shown a significant potential for mask-less fabrication of well-defined micro- and nanostructures, which turned out to be inexpensive, precise, and, in some cases, efficient in repelling cells and bacterial colonization [2,8,27,29]. Hierarchical micro/nanostructures that control the cells’ attachment and migration have been developed by direct laser irradiation of various polymeric and metallic surfaces [8,27]. Among these, LDW via TPP has been found suitable for producing complex 3D polymeric structures able to guide cellular behavior. For example, Klein et al. employed LDW via TPP to produce 3D scaffolds at a micrometric scale using two different photopolymers, which controlled the cell adhesion in 3D [19]. Richter et al. used LDW via TPP to control the chemical properties of 3D micro-scaffolds [20]. Claus et al. performed a simultaneous orthogonal functionalization of 3D microstructures by LDW via TPP of two photopolymers with photo-reactive groups [21]. Richter et al. prepared a 3D micro-scaffold by combining a protein repellent photoresist with a protein adhesive and a photo-activated passivated adhesive for bioactive functionalization of two extracellular matrix proteins on the scaffolds [30].

In this context, in the present study, we designed and fabricated innovative hierarchical structures by LDW via TPP of a photopolymerizable standard formula named IP-Dip. The laser-generated structures (Figure 2) closely followed the design (Figure 1). The mushroom-like constructs consisted of micro-posts (mushrooms legs) sustaining horizontal surfaces (mushrooms hats) having micro- and nanostructures on top. A ripple-like pattern was created on top of the mushrooms, over length scales ranging from several µm in height and width down to tens of nm in height and hundreds of nm in width (Figure 3).

### 2.3. Contact Angle Measurements

**Figure 5:**
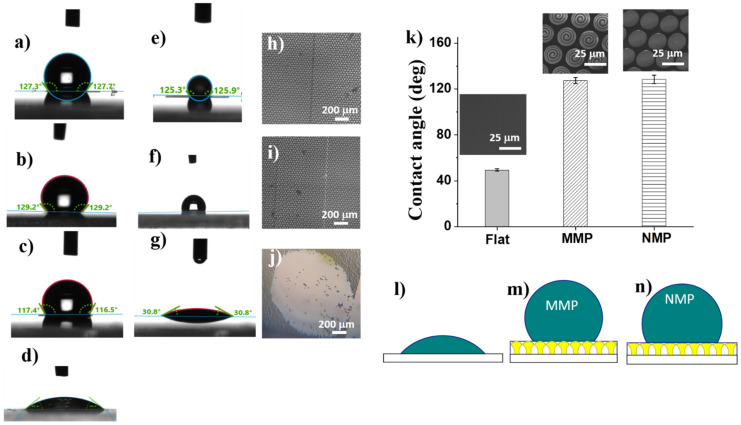
Water drops on: (**a**) microstructured mushroom-like pillars (MMP); (**b**) nanostructured mushroom-like pillars (NMP); (**c**) mechanically instable NMP; (**d**) flat dry polymer surface; (**e**–**g**) water drops on corresponding surfaces from (**a**–**c**) after drying for 10 min; (**h**–**j**) optical microscopy images of surfaces from (**a**–**c**) after complete drying of the water drop; (**k**) contact angles and scanning electron micrographs on the surfaces from (**a**,**b**,**d**); (**l**–**n**) schematic illustration of water drop on flat surface, microstructured mushroom-like pillars (MMP) and nanostructured mushroom-like pillars (NMP). Laser fabrication parameters: (**h**,**i**) laser speed 100 µm/s, laser power 12.5 mW; (**j**) laser speed 120 µm/s, laser power 12.5 mW.

To evaluate the wettability of the MMP and NMP structures, the water contact angles between water drops and MMP and NMP surfaces, respectively, were measured (Figure 5). Both MMP and NMP structures exhibited significantly increased contact angles as compared to the flat polymer surface, which turned out to be hydrophilic (Figure 5d). The hydrophobic character of MMP and NMP structures was induced by a certain level of surface roughness since most probably the mushroom-like structures entrap the air underneath and in between.

To find out the reasons for the hydrophobic character of the NMP and MMP structures, we looked in more detail into the surface morphology of the samples, as revealed by SEM and AFM images from Figure 3. Although the scales of the structuring differed by 10 orders of magnitude, i.e., from micrometers for MMP to nanometers for NMP, the surface roughness of both MMP and NMP structures seemed to be sufficient to achieve hydrophobicity. The wettability changed from hydrophilic on flat polymer surfaces, having the contact angle of (43 ± 1)° (Figure 5d), to hydrophobic on MMP and NMP surfaces, which showed contact angles of (127 ± 2)° and (128 ± 4)° respectively (Figure 5a,b). Most likely, the mushroom-like structures trapped air underneath the mushrooms’ hats while limiting the penetration of water, as schematically illustrated in Figure 5l–n.

Given the mechanical instability observed for some of the hierarchical structures from Figure 2a, we checked if the wettability changed in time [28] by investigating the temporal stability of water drops on hierarchical structures fabricated using various laser parameters. The MMP structures fabricated using 120 µm/s laser scanning speed, and 12.5 mW laser power collapsed under the weight of the water drop (Figure 5j) that was spread on the surface leading to a low contact angle of 310 (Figure 5g). This behavior can be associated with the mechanical instability of the MMP structures evidenced in Figure 2b. Optical microscope images recorded after the drying of the water drop showed that the mushroom-like structures were washed away from the glass substrate (Figure 5j). The hierarchical structures that were mechanically stable remained hydrophobic the whole time, which took a drop of water to dry, i.e., about 10 min (Figure 5e,f), and preserved their place on the glass substrate (Figure 5h,i). The difference between the stable and unstable structures is related to the laser processing parameters used for structure fabrication (laser power and laser scanning speed), as specified in the caption of Figure 5.

Numerous studies indicate that the wettability of a surface can be changed by varying the feature size, which in turn modifies the contact area between the surface and the droplet. Moreover, it has been shown that surface wettability can be tuned by patterning the surface with micro- and nano-topographies, such as pillars, pits, channels, and ridges [31]. The ability of such micro and nano-features to induce different wetting behaviors for a surface has been used for controlling the surface adhesion of diverse biological species [32].

In a hierarchical topography, the air entrapment and the subsequent settlement of cells were found to depend on the balance of micro- and nanoscale structures [33,34,35]. Li et al. described that hierarchical wrinkles on a polymer substrate result in higher contact angles when compared to surfaces with random wrinkles [34]. There are two models employed for describing different wettability behaviors of a surface [21]. One is the “Wenzel” mode, according to which the interface between the surface and water droplet increases with increasing surface roughness. As a result, for hydrophobic or hydrophilic surfaces, the water contact angle increases or decreases, respectively. The other model is called Cassie- Baxter regime and applies when the surface features become smaller and closer packed [6]. In this case, the air enclosed between the surface and the water droplet reduces the contact between the surface and the droplet, and the latter comes into contact only with the upper parts of the surface features. This model seems to apply well to the hierarchical NNM and MMP structures that we developed. The air enclosed between the laser-patterned surface and the water droplet minimized the contact area, and the hydrophobicity was increased by enhancing the surface roughness. According to [6], the apparent contact angle (θ_CB_) is given by:

cosθ_CB_ = f_solid_(cosθ_E_ + 1) − 1 (3)

where cosθ_E_ is the contact angle measured on the flat polymer surface and f_solid_ is the percent of the water droplet that is in contact with a solid surface. Therefore:

f_air_ = 1 − f_solid_ (4)

represents the fraction of the water droplet that comes into contact with the air entrapped by the nano- and micro-features from the surface instead of the solid surface.

Indeed, from the results synthetized in Table 2, most of the water droplets came into contact with the air entrapped within the nano- and microstructures decorating the mushrooms’ hats (76% for NMP and 77% for MMP), explaining the hydrophobic character of these structures.

Moreover, recent studies mention that the water droplets touch the peaks exclusively from the laser-patterned surface, where they give rise to more liquid-air interfaces than an entire liquid-solid interface [36]. For hierarchical structures, the enhancement in water repellence has been assigned to the dual-scale surface roughness [36,37]. These considerations explain well our experimental observations, and we may conclude that the hierarchy of the structures fabricated by LDW via TPP provided sufficient air-trappable sites to make the MMP and NMP structures hydrophobic.

### 2.4. Biological Assays

The in vitro studies were conducted on OLN-93 cells, which is an oligodendroglia cell line derived from primary rat brain glial cultures. Oligodendrocytes are part of a network of interconnected glial and neuronal cells, with an important role in providing physical and metabolic support for neurons, responding to neural activity, and regulating the homeostasis of water and ions [38,39]. The OLN-93 cell line is highly reactive to the environment to which it expresses morphological changes, depending on the stage of cell differentiation. These cells emit phyllopodia through which they attach to the surroundings; in vivo, the phyllopodia attach to the axons of neuronal cells, where they form the myelin sheaths [40,41].

**Figure 6:**
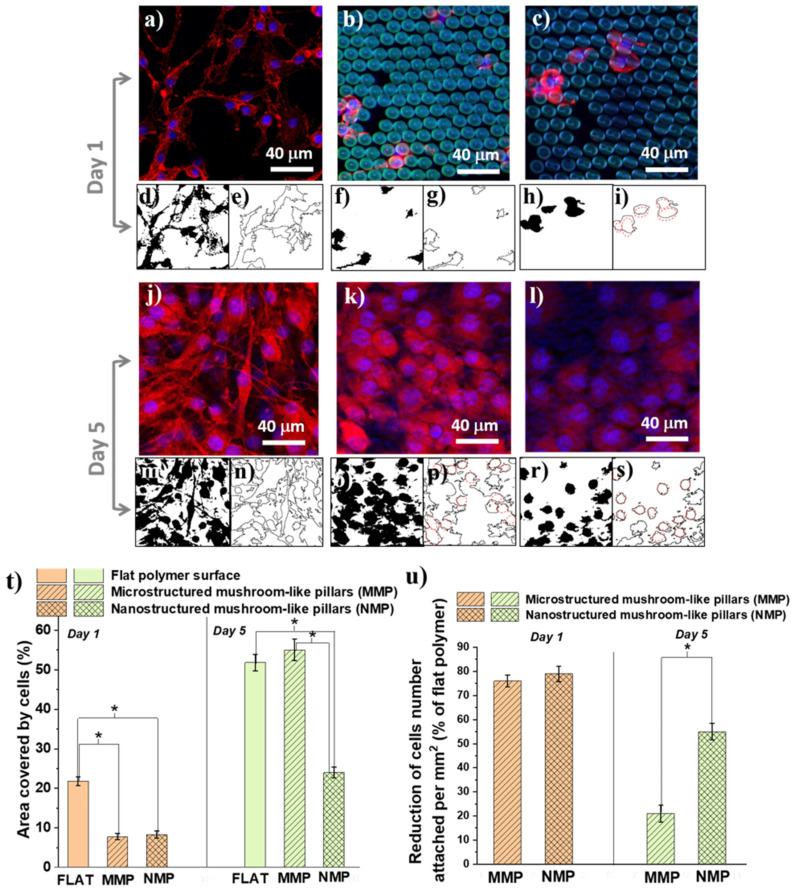
Representative fluorescence microscopy images of OLN-93: (**a**,**j**) flat polymer surface; (**b**,**k**) microstructured mushroom-like pillars (MMP); (**c**,**l**) nanostructured mushroom-like pillars (NMS), after one and 5 days of cell culture. Colors: cytoskeleton: F-actin (red); nuclei: Hoechst-blue; structures’ autofluorescence-green. (**d**,**f**,**h**,**m**,**o**,**r**) black-and-white masks identifying the cells on surfaces from (**a**–**c**,**j**–**l**) after one and 5 days of cell culture; (**e**,**g**,**i**,**n**,**p**,**s**) contour lines indicating the outline of each cell from (**a**–**c**,**j**–**l**), after one and 5 days of cell culture; (**t**) Area covered by cells as % from 1 × 1 mm^2^ surface after one and 5 days of cell culture; (**u**) Cells number per 1 × 1 mm^2^ surface, calculated as percent of flat polymer surface (control), after one and 5 days of cells culture. * corresponds to *p* ≤ 0.05 as specified in the Section 3.5.

The cells were immunostained with F-actine (cytoskeleton) and Hoechst (cells nuclei) after one day (Figure 6a–c) and 5 days (Figure 6j–l) of cell culture. The fluorescence images were evaluated in terms of cellular shape (Figure 6d–i,m–s), the area covered by cells (Figure 6t), and reduction in cell number density per mm^2^ relative to a flat polymer surface (Figure 6u).

Concerning the cellular shape, we observed that, irrespective of the culture time, the cells from the flat polymer surfaces had a natural spindle-like shape with phyllopodia (Figure 6d,e). At early times of cell culture, on the MMP structures, the cells retained their native morphology, even though they were less numerous than on the flat surface (Figure 6f,g). On the NMP structures, the cells dramatically changed their shape form spindle-like with phyllopodia to a round shape with almost no protrusions (Figure 6h,i, where the red outlines from Figure 6i evidence the round shape of the cells). After 5 days of cell culture, the cells from both MNP and MMP structures changed their shape and became round with no phyllopodia (Figure 6o–s).

Next, we measured the areas covered by cells for each type of hierarchical structure in comparison with a flat polymer sample of the same size (control) 1 × 1 mm^2^ surface (Figure 6t). After 24 of cell culture, there was no statistical difference between MMP and NMP structures, with a total area covered by cells between 7% and 8%. On flat surfaces, at this early stage of cell culture, a much higher percent, i.e., almost 22%, was covered by cells. After 5 days of cell culture, the flat surfaces and the MMP structures were covered by cells in a proportion of about 52–54%, with no statistical difference between them. The NMP structures reduced the cells-covered area down to 24%. Regarding the number of attached cells (Figure 6u), we observed that in the first 24 h, both MMP and NMP structures lowered the cellular attachment as compared to flat polymer surfaces (controls). Namely, the cellular attachment was reduced by 76% for MMP and 78% for NMP structures, as compared to the control. After 5 days of incubation, the reduction in the cell attachment on MMP structures was only 21% as compared to the control, while the NMPs structures showed a much better cell-repellent efficiency, reducing the cell attachment by 55% as compared to the control.

Our studies indicated that the cells attached in different ways on flat and MMP and NMP hierarchical structure. The cells from the flat surfaces were stretched and had a spindle-like shape with phyllopodia. At longer incubation times, the cells formed aggregates over hundreds of μm. A similar trend was observed for the MMP structures, with fewer cells attached. In contrast, on the NMP structures, the cells attached to a much lower extent showed a round shape, and their coalescence was inhibited. These findings clearly indicate that surface nanostructuring strongly hindered cellular adhesion. On the opposite, the ripple-like features from the MMP structures were of 3 orders of magnitude higher than the typical thickness of cells phyllopodia, explaining the much weaker influence of these structures on the cells’ shape and adhesion. In a first approximation, these in vitro results are representative of other cell types. In particular, the cells’ behavior on NMP structures can be ascribed to the fact that the quasi-periodicity of the ripple-like features that decorate the mushrooms’ hats was of hundreds of nm (as shown in Figure 3), which is a typical size range for the cells phyllopodia. The validity of our experimental findings concerning other cell types is supported by the fact that the periodicity of the nanostructures from NMPs is comparable with the typical thickness of the cytoplasmatic phyllopodia that link a cell to a substrate, which is in (60–200) nm interval [4].

**Figure 7:**
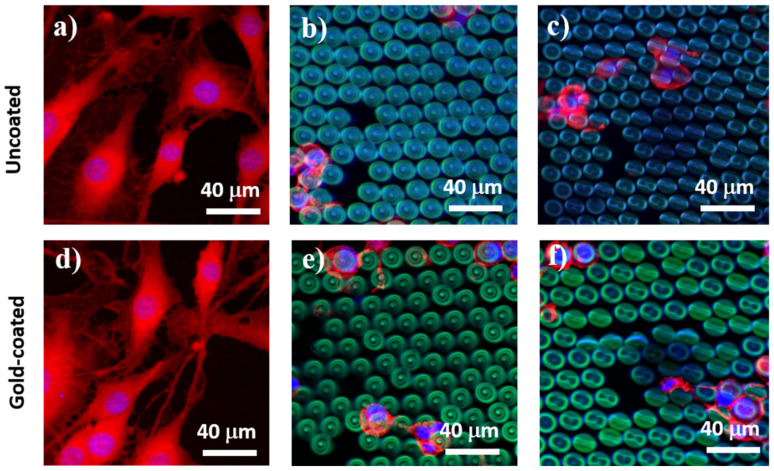
Representative fluorescence microscopy images of cells cultured for 24 h on: (**a**,**d**) flat polymer surface; (**b**,**e**) microstructured mushroom-like pillars (MMP); (**c**,**f**) nanostructured mushroom-like pillars (NMS). Cytoskeleton: F-actin (red); nuclei: Hoechst (blue); structures’ autofluorescence (green). Upper panel: uncoated samples. Lower panel: samples coated with 14 nm layer of gold.

To separate the role of topography from the role of wettability, we repeated the in vitro experiments on flat and on NMP and MMP structures after each of them was coated with a 14 nm layer of gold. The basic idea behind this test was to change the structures’ wettability while preserving their topography. After the gold coating, the contact angles measured on the MMP and NMP structures decreased considerably as compared to the uncoated structures. More precisely, the contact angles decreased from about 123° (as measured on uncoated MMP and NMP structures) to about 33° measured for the gold-coated MMP and NMP ones. As expected, the thin gold layer did not change the 3D architecture of the structures, as it could be easily observed by SEM investigations. Instead, it only turned the MMP and NMP structures from hydrophobic to hydrophilic. Interestingly, the cellular behavior on the gold-coated MMP and NMP structures was the same as for the uncoated MMP and NMP samples, with the only exception that on the former, there were slightly more cells attached (Figure 7). This is in fair agreement with recent studies indicating that, at the early stages of cell culture, the low level of surface energy characteristic of hydrophobic substrates delays the cellular attachment [6]. The fact that the cellular shape on the gold-coated samples followed the same trend as for the uncoated samples (changing from spindle-like with protrusions on flat substrates to round shape without protrusions on MMP and NMP structures) indicates that the cellular behavior was mainly governed by structures’ topography rather than by their wettability.

The reason for which in Figure 7 we showed cells at 24 h of cell culture and not for longer culture time is because the only role of this test was to observe the cells from uncoated versus gold-coated structures in order to distinguish between the influence of the topography from that of the wettability. If we would have extended the cell culture time interval, then the cells would have agglomerate on the structures, and this would have impeded a sharp observation of cell morphology. One can state that, from the morphological point of view, the cells at later stages, e.g., 5 days of culturing, look similar to the cells from Figure 6j–l, providing thus no relevant information regarding the tests on uncoated versus gold-coated structures from Figure 7.

It has been demonstrated that aside from cell growth, division, and death, the changes in cellular shape are one of the main factors involved in tissue morphogenesis [42]. Because of the complexity of the issue, the precise mechanisms beyond this influence remain unrevealed. Until present, this topic has been addressed by theoretical modelings, such as in bottom-up approaches determining how the shape change in an individual cell controls the tissue morphogenesis, or by top-down methods investigating how tissue morphogenesis is related to cell shape changes [42]. A particular cell property is the cell polarity, which is related to the asymmetric organization of cellular components, such as plasma membrane, cytoskeleton, or organelles. However, the investigation of every one of these aspects implies sophisticated bio-molecular investigations and is not in the scope of the present study.

**Figure 8:**
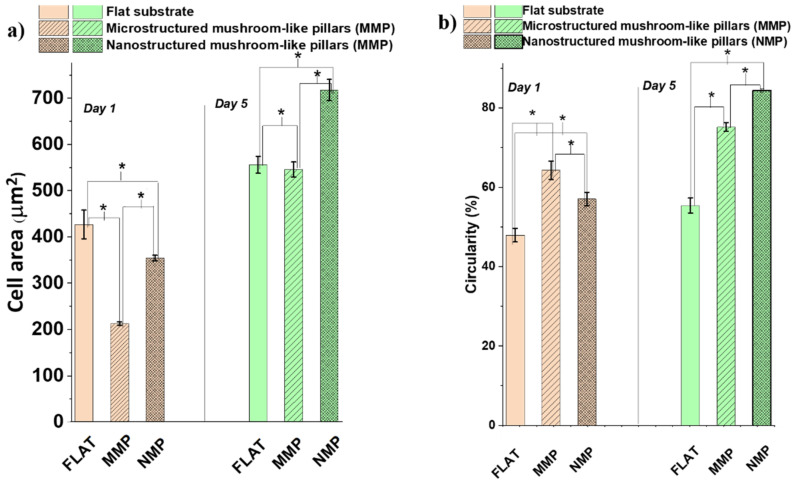
(**a**) Cell area and (**b**) circularity of cells cultured on: flat surfaces, microstructured mushroom-like pillars (MMP), and nanostructured mushroom-like pillars (NMP), after 1 and 5 days of cell culture. The measurements were performed using fluorescence images from Figure 6 and Image J software. A circularity of 100% corresponds to a circle. * corresponds to *p* ≤ 0.05 from the Section 3.5.

To gain some insight into the changes induced by the interaction of the cells with the hierarchical structures, we looked into more detail to the morphology changes that could possess biological significance for the function of the cells, such as surface area per cell and shape of the cytoskeleton as particular aspect related to cells polarity. These were evaluated quantitatively by measuring the cell area (Figure 8a) and circularity (Figure 8b). Within the first 24 h of cell culture, the cells from both MMP and NMP hierarchical structures were much less spread than the cells from the flat substrates. For example, the area per cell on MMP structures decreased by almost 50% as compared to the area of a cell seeded on a flat substrate (Figure 8a). After 5 days of cell culture, the cells from the flat and MMP structures showed similar areas per cell, whereas the NMP structures induced an increase in the cell area with almost 25%. To relate these changes, at least tentatively, to biological functions of the cells, the observations regarding the area per cell must be mandatorily correlated with quantitative evaluations of the cellular shape, which represents a “first view” indicator of cell differentiation [43]. To investigate quantitatively how much the cellular shape deviates from the native, polygonal shape of the cells as, for example, seen for cells cultured on flat substrates, Figure 8b presents measurements of the cells’ circularity. A circularity of 100% was considered to correspond to a perfect circular shape. The measurements confirm the qualitative observations from Figure 6, proving that the cells from both MMP and NMP hierarchical structures lost their native, polygonal shape observed for the flat substrates. Namely, the cells from both MMP and NMP structures had a high percentage circularity for all culture time intervals investigated. The cells became almost round and had much fewer protrusions (as indicated by the higher percentage of circularity for these samples). These changes became more evident for the cells cultivated on NMP structures and for longer culture time intervals. As it is known that the cellular shape is related to the degree of cell differentiation [43,44,45], we can advance the preliminary conclusion that both MMP and NMP hierarchical structures drastically changed the area and shape of OLN-63 cells and that the more pronounced changes were induced by the NMP structures and for longer culture time intervals. Most likely, the hierarchical structures provided poor adhesion points for the cells that were not allowed to spread along the structures and to differentiate to an arborized, i.e., with extensions cellular morphology. Further investigations regarding the morphological and functional differentiation of the OLN-93 cell line are beyond the scope of the present study and will be the subject of further research. At this point of the experiments, the OLN-93 cell line was used as a cellular model for investigating the antiadhesive properties of the hierarchic structures fabricated by laser direct writing.

The response of glial cells to hierarchic 3D architectures at two distinctly (micro- and nano-) length scales was investigated. Our experimental results indicated that the NMP structures had a stronger cell-repellent effect than the MMP structures, which was more obvious at a long time of cell culture (Figure 5k). Interestingly, this occurred even though both NMP and MMP hierarchical structures were equally hydrophobic. This finding is in agreement with other studies reporting that surface topography plays a more important role in cellular adhesion than surface hydrophobicity [6]. Moreover, it has been shown that cell adhesion and spreading are more inhibited on laser-patterned nanopatterned substrates than on micropatterned substrates [6,8]. The microscale topographical cues were considered less able to impede the cellular attachment because they are far from the length scale of focal contacts (e.g., ~100–200 nm depending on cell maturity) [12,42]. On the opposite, the nanostructures appear to reduce the cell adhesion, which was attributed to the cells’ inability to spread along water repellent surfaces as well as to the role of nanoscale structuring that reduced the contact area between cells and the solid surface [42]. The mechanism of cell adhesion has also been correlated with key roles played by cell phyllopodia [44,45]. Similar to our experimental findings, studies on neuronal cells have shown that on flat surfaces, the cells were stretched and formed aggregates, whereas on nanoscale ripple-like features, the cells were round, and their coalescence was inhibited [46].

Overall, it appears that the influence of the micro and nano-roughness of the MMP and NMP hierarchical structures dominated the effect of surface wettability and led to a much smaller contact area between the cells and the underlying surface [6]. The lack of affinity between the cells and the MMP and NMP hierarchical structures can be ascribed to the fact that the micro- and nanopatterns that decorate the mushrooms’ hats have poor matching with the complex irregular morphology of the cells [4]. Some studies evidenced that wettability is also involved in the cell-substrate interactions, but the optimum value of the contact angle for cell attachment and spreading remains unknown. For example, hydrophobic surfaces were found to impede the attachment of small units, whereas large units like the cells were not necessarily restricted by surface hydrophobicity [47].

In the present study, we investigated the response of oligodendroglia cell line OLN-93 to hierarchical micro/nano-topographies. The choice of this particular cell type was based on the fact that OLN-93 is a cell line that provides a useful model system for the investigation of specific mechanisms regulating the proliferation and differentiation of oligodendrocytes in vitro [48]. The experimental findings of the present work provide a starting point that opens up interesting perspectives for further evaluations on other cell lines as well. In the nearest future, we envisage addressing the interactions of the hierarchical structures with other cells of the nervous system, such as neuro-2a neuroblastoma cells.

Different research groups have reported a strong correlation between substrate roughness and the adhesion modifications in different types of cells. For example, the influence of substrate morphology on neuronal cells [48], the role of nanopores on mesenchymal stem cells [49], and the effect of the diameters of TiO_2_ nanotubes on the adhesion and spreading of mesenchymal cells [46] have been discussed. It is generally recognized that the cells phyllopodia play key roles in the mechanism of cell adhesion on a substrate [45]. Specifically, it has been shown that when the phyllopodia are tilted with respect to the ripples-like structures, the contact occurs only in few points, and the cellular adhesion is diminished [4]. To address this issue, in future studies, we will pursue investigations concerning the response of the phyllopodia of the OLN-93 cell line as well as of other cells of the nervous system to hierarchical topographies. Nevertheless, we should keep in mind that the cell-substrate interactions are quite complicated by the fact that the phyllopodia are not rigid [4].

## 3. Materials and Methods

### 3.1. Design of Hierarchical Structures

The design of the structures was based on a spiral-like writing method. In order to define the structure using Cartesian coordinates, an algorithm was developed in Python 3.5. The Python script requires a series of parameters as input from the user (height, diameter, line step, etc.) and then generates all the points that define a given structure. This list of points is then written in a file, along with several specific commands for the 3D lithography installation, which can be loaded in the control software of the installation. The script also generates a scaled 3D plot in order to verify if the geometric characteristics of the structure are appropriate for the intended application and fabrication method.

### 3.2. Fabrication of Hierarchical Structures

The structures were fabricated using the 3D lithography installation from Nanoscribe (Photonic Professional). This is an installation specific for laser direct writing technique based on two-photon polymerization (LDW via TPP). The laser source was an Er-doped fiber laser delivering 120 fs pulses with a repetition rate of 80 MHz, a maximum average power of 120 mW, centered on a wavelength λ = 780 nm. Laser pulses were directed to the photopolymerizable material through an inverted microscope. The focusing of the laser beam was performed using a 100×, NA = 1.3 microscope objective. For structure fabrication, we used IP-Dip photopolymer, an acrylic-based liquid photoresist that requires no pre- or post-processing and that is optimized for the 3D lithography system Nanoscribe GmbH. The photopolymer was drop-casted on a SiO_2_ substrate. The laser writing process was achieved by immersing the microscope objective in the photopolymer itself. Sample positioning was performed using two sets of XYZ translation stages: a step-by-step set with ±1.5 µm accuracy for general movement and a piezo set for high accuracy positioning. Following laser irradiation, the unpolymerized material was removed by immersing the samples in propylene glycol methyl ether acetate for 10 min, followed by gentle washing with isopropanol.

### 3.3. Investigations of Hierarchical Structures

#### 3.3.1. Scanning Electron Microscopy

Morphological information about the hierarchical structures was obtained through scanning electron microscopy (SEM). For this, we used a Quanta Inspect F50 system, which has a field emission gun (FEG) with a 1.2 nm resolution. The SEM equipment allowed us to minimize the adjustments to the column when changing kV. All adjustments were carried out automatically upon the selection of kV and/or spot size settings. For the hierarchical structures investigated in this study, the images were recorded at 30 kV accelerating voltage. The probe current was continuously adjustable up to 2 μA. For better observation of the architecture of the hierarchical structures, in some cases, the samples were tilted at 37 degrees. Prior to SEM analysis, the structures were coated for 60 s with a thin gold film (thickness of several nm).

#### 3.3.2. Atomic Force Microscopy

Micro- and nanoscale morphology measurements of the hierarchical structures were conducted on a commercial atomic force microscopy system (AFM, XE100—Park Systems, Suwon, Republic of Korea). The measurements were performed in non-contact mode, using standard cantilevers (AC240TS—Olympus Corporation, Tokyo, Japan; NCHR—Nanosensors, Neuchatel, Switzerland).

#### 3.3.3. Contact Angle Measurements

Static water contact angle measurements (sessile drop shape analysis) of the samples were performed using a Krüss DSA 100 Drop Shape Analyzer goniometer equipped with a camera and recording system. The measurements were carried out using ≤1 μL ultrapure water (θ_water_) drop placed on the surface and recorded for 10 s. The needle size was G27. The average contact angle was calculated from 20 measurement points (2 frames/second from the recorded video) of one drop using the circle fitting as a computing method.

### 3.4. Investigations of Hierarchical Structures

#### 3.4.1. Cells Seeding

OLN-93 used for tests in these experiments is an oligodendroglia cell line from spontaneously transformed cells in primary rat brain glial cultures [50]. OLN-93 cells were provided by the University of Hasselt, Belgium, with the agreement of the Department of Biology, Molecular Neurobiology, University of Oldenburg, Germany. The cells were grown in an incubator (SafeGrow Pro—Euroclone) under standard conditions (37 °C, 5% CO2 and ~95% relative humidity) in 25 cm^2^ flask (TPP catalog number 90028), in growth medium (DMEM, Sigma-Aldrich catalog number D6429, St. Louis, MO, USA) supplemented with 10% Fetal Bovine Serum (Merck catalog number 1233C, Darmstadt, Germany) and 0.5% Penicillin-Streptomycin (Thermo Fisher catalog number 15070063, Waltham, MA, USA). Prior to cell-seeding, the samples were sterilized for one hour under an ultraviolet lamp (Sankyo Denki G30T8). The cells were detached with Trypsin-EDTA solution (Sigma-Aldrich catalog number T2610), re-suspended in the growth medium, counted, and seeded on the NMP and MMP hierarchical structures. All procedures that required sterile work were performed in microbiological safety cabinet Safeflow 1.2 (Euroclone). After 24 h, the growth medium was replaced with differentiation medium (growth medium with 0.5% FBS) [51].

#### 3.4.2. Cells Staining

After one day and 5 days of incubation, respectively, the cells attached to the hierarchical structures were labeled with Texas Red™-X Phalloidin (Thermo Fisher catalog number T7471) for cytoskeleton and Hoechst 33,342 (Thermo Fisher catalog number H1399) for nuclei. A special protocol was developed prior to this step: the cells were washed twice with phosphate-buffered saline solution (PBS), fixed with 3.7% paraformaldehyde (Chemical Company) for 30 min, permeabilized 10 min with 0.1% Triton X-100 (Merck catalog number X100-100ML), incubated for three hours at room temperature with Texas Red™-X Phalloidin, washed again with PBS and finally incubated with Hoechst for 10 min. The fluorescence images were acquired using an Olympus BX 51 microscope equipped with a DSD2 module with a pE-4000 light source and Olympus UPLFLN 40× objective. For microscope image acquisition and control software, we used Andor IQ3.

#### 3.4.3. Quantification of Cells Shape, Number, Cell Area, and Circularity

Quantitative information regarding the cell number density, cell total area, area per cell, and cell circularity was obtained using ImageJ v1.36. The number of cells and the cell area per surface area were calculated using the “Analyze particles” plugin. For this, the images obtained by fluorescence microscopy were transformed into 8-bit black-and-white images and thresholded (to emphasize the entities to be analyzed, i.e., the cells). The range for “pixels^2” was set from 0 to infinity, and the circularity was set from 0 to 0.8. The results were set to “Mask” (where the cells were black and the exterior was white) and to “Bare outlines” (displaying the contour of each cell). The results represent the means of 5 different experiments (in total 5 fields of view for each sample).

### 3.5. Statistical Analysis

The data points were expressed as mean ± standard deviation. The statistical analysis was performed using Student’s test to determine differences between groups (* corresponded to *p* < 0.05). Data from 5 different experiments performed in identical conditions were used.

## 4. Conclusions

An appropriate combination laser power-scan speed, “accompanying” a dedicated written software, allowed us to obtain polymeric hierarchical mushroom-like structures by laser direct writing via two photons polymerization of IP-Dip photopolymer. The structures were organized in hexagonal lattices, with a “mushroom” total height of 18 µm, of which 15 µm was the supporting underside structure (mushroom’s leg), and 3 µm was the top side (mushroom’s hat), each of these parts having different parabolic curvatures. The mushrooms’ hats were decorated with micrometric or nanometric quasi-circular ripple-like features. The obtained structures were denominated microstructured mushroom-like pillars MMP and nanostructured mushroom-like pillars NMP, respectively. The widths of the ripple-like structures from MMP ranged between 2 and 6 μm, and their heights were about 2.5–3 μm, with calculated aspect ratios between 1.3 and 1.6. The ripple-like structures from NMP were between 60 and 170 nm in height, their width ranged between 440 and 740 nm, and the calculated aspect ratios were between 3.8 and 7.2. Both MMP and NMP structures were hydrophobic, with contact angles of (127 ± 2)° and (128 ± 4)° respectively, whereas the flat polymer surfaces were hydrophilic, i.e., the contact angle of (43 ± 1)°. The response of glial cells to the interaction with the MMP and NMP hierarchical structures evidenced important characteristics depending on the two distinctly (micrometric and nanometric) length scales. In the first 24 h, the cellular attachment was reduced by 76% for MMP and 78% for NMP structures, as compared to the control. After 5 days of incubation, the reduction in the cell attachment on MMP structures was only 21% as compared to the control, while the NMPs showed better cell-repellent efficiency, reducing the cell attachment by 55% as compared to the control. In addition, the NMP structures changed the normal cellular shape (spindle-like with phyllopodia, encountered on flat and MMP structures) to a round shape with no protrusions and inhibited the cells’ coalescence. The influence of structures’ wettability on cellular adhesion and morphology was found to be less significant. We may conclude that the cellular behavior was mainly driven by structures’ topography and that the nanostructured mushroom-like pillars (NMP) showed higher cell-repellent efficiency than the flat and microstructured ones (MMP).

## Figures and Tables

**Table 1:** Heights, periodicities, and aspect ratios of the ripple-like features from the top of the mushroom-like structures fabricated with different laser writing parameters and measured from the line profiles in Figure 3.

	Laser Speed Laser Power	Height (nm)	Width (nm)	Aspect Ratio
	140 µm/s 13.8 mW	Not measurable *	(1734–2857)	-
**MMP**	120 µm/s 12.5 mW	(2540–2705)	(3440–3960)	(1.3–1.6)
	100 µm/s 12.5 mW	(2570–3800)	(3776–5667)	(1.4–1.5)
	140 µm/s 13.8 mW	Smooth surface	Smooth surface	-
**NMP**	120 µm/s 12.5 mW	(69–172)	(490–740)	(4.3–7.2)
	100 µm/s 12.5 mW	(116–154.6)	(440–529.3)	(3.4–3.8)

* (incomplete excursion of AFM needle on the vertical axis, i.e., it did not reach the bottom of the periodical microstructures).

**Table 2:** Water contact angles measured on MMP and NMP hierarchical structures according to Cassie-Baxter model (θ_CB_) and on flat polymer surface (θ_E_), the water droplet fraction coming in contact with the air entrapped within the nano and micro-features from NMP and MMP structures (f_air_) and water droplet fraction that is in contact with the solid surface (f_solid_), calculated assuming a Cassie-Baxter model.

Type of Hierarchical Structure	θ_CB_ (°)	θ_E_ (°)	f_solid_ (%)	f_air_ (%)
MMP	127 ± 2	49 ± 1	24	76
NMP	128 ± 4	49 ± 1	23	77
